# Comparison of [^18^F]PSMA-1007 with [^68^Ga]Ga-PSMA-11 PET/CT in Restaging of Prostate Cancer Patients with PSA Relapse

**DOI:** 10.3390/cancers14061479

**Published:** 2022-03-14

**Authors:** Manuela A. Hoffmann, Finn Edler von Eyben, Nicolas Fischer, Florian Rosar, Jonas Müller-Hübenthal, Hans-Georg Buchholz, Helmut J. Wieler, Mathias Schreckenberger

**Affiliations:** 1 Department of Occupational Health & Safety, Federal Ministry of Defense, 53123 Bonn, Germany; 2 Clinic of Nuclear Medicine, Johannes Gutenberg University, 55101 Mainz, Germany; hans-georg.buchholz@unimedizin-mainz.de (H.-G.B.); mathias.schreckenberger@unimedizin-mainz.de (M.S.); 3 Center for Tobacco Control Research, DK-5230 Odense, Denmark; finn113edler@mail.tele.dk; 4 Department of Urology, University of Cologne, 50937 Cologne, Germany; nicolas.fischer@uk-koeln.de; 5 Department of Nuclear Medicine, Saarland University Medical Center, 66421 Homburg, Germany; florian.rosar@uks.eu; 6 Practice of Radiology and Nuclear Medicine, Praxis im KölnTriangle, 50679 Cologne, Germany; jmh@praxis-im-koelntriangle.de; 7 Medical Center, University of Dusseldorf, 40225 Dusseldorf, Germany; helmut.wieler@web.de

**Keywords:** [^18^F]PSMA-1007 PET/CT, [^68^Ga]Ga-PSMA-11 PET/CT, prostate-specific antigen, prostate cancer, PSA threshold level, optimal PSA cut-off level, biochemical recurrence

## Abstract

**Simple Summary:**

Prostate cancer (PC) is one of the most common malignancies in men. Prostate-specific membrane antigen (PSMA) positron emission tomography (PET) hybrid imaging can help improve the diagnosis of recurrent PC, in addition to conventional methods such as computed tomography (CT) or magnetic resonance imaging alone. In order to further evaluate the importance of PSMA hybrid imaging, this study identified prostate-specific antigen (PSA) threshold levels for the detection of PSMA-positive lesions in patients with elevated PSA values after therapy. The thresholds were calculated for two different radiopharmaceuticals, PET/CT with [^18^F]PSMA-1007 and [^68^Ga]Ga-PSMA-11, and the results were then compared. In patients who had previously undergone prostatectomy, there was an advantage of PET/CT with [^18^F]PSMA-1007. Overall, however, the results of both methods were similar. The findings may help improve PC detectability in the future and are likely of considerable interest to clinicians by refining algorithms to help determine the clinical approach for the evaluation and treatment of patients with elevated PSA values after therapy.

**Abstract:**

This study aimed to compare the diagnostic performance of [^18^F]PSMA-1007 positron emission tomography/computed tomography (PET/CT) (^18^F-PSMA) and [^68^Ga]Ga-PSMA-11 PET/CT (^68^Ga-PSMA) by identifying prostate-specific antigen (PSA) threshold levels for optimal detecting recurrent prostate cancer (PC) and to compare both methods. Retrospectively, the study included 264 patients. The performances of ^18^F-PSMA and ^68^Ga-PSMA in relation to the pre-scan PSA were assessed by receiver operating characteristic (ROC) curve. ^18^F-PSMA showed PC-lesions in 87.5% (112/128 patients), while ^68^Ga-PSMA identified them in 88.9% (121/136). For ^18^F-PSMA biochemical recurrent (BCR) patients treated with radical prostatectomy (78/128, patient group: F-RP), a PSA of 1.08 ng/mL was found to be the optimal cut-off level for predicting positive and negative scans (AUC = 0.821; 95%, CI: 0.710–0.932), while for prostatectomized ^68^Ga-PSMA BCR-patients (89/136, patient group: Ga-RP), the cut-off was 1.84 ng/mL (AUC = 0.588; 95%, CI: 0.410–0.766). In patients with PSA < 1.08 ng/mL (F-RP) 76.3% and <1.84 ng/mL (Ga-RP) 78.6% scans were positive, whereas patients with PSA ≥ 1.08 ng/mL (F-RP) or 1.84 ng/mL (Ga-RP) had positive scan results in 100% and 91.5% (*p* < 0.001/*p* = 0.085). The identified PSA thresholds for PSMA-mappable PC lesions in BCR-patients (RP) showed a better separation for ^18^F-PSMA with regard to the distinguishing of positive and negative PC-lesions compared to ^68^Ga-PSMA. However, the two PSMA PET/CT tracers gave similar overall findings.

## 1. Introduction

In Europe and the United States, prostate cancer (PC) is one of the most common solid tumors in men [1,2]. The mortality varies 10-fold depending on the pathological risk category [3]. Patients with localized PC are usually treated with some combination of surgery and/or radiotherapy and/or other local strategies, while treatment options for patients with advanced PC may include hormone therapy, androgen deprivation, chemotherapy and radionuclide therapy (using radiolabeled tracers) [4]. For patients previously treated with radical prostatectomy (RP) biochemical recurrence (BCR) is defined as a prostate-specific antigen (PSA) > 0.2 ng/mL after RP, which is confirmed in at least two measurements. For patients initially treated with radiotherapy (RT) BCR is defined as a PSA > 2 ng/mL above the PSA nadir after the RT [5].

In recent years, the early detection of primary PC as well as the detection of PC recurrences and distant metastases have been significantly improved by metabolic imaging methods [6,7]. In patients with suspected PC recurrence after initial intended curative treatment, diagnostic imaging is a challenge for assessing tumor recurrence or distant metastases. The current standard imaging methods include transrectal ultrasonography (TRUS), computed tomography (CT), magnetic resonance imaging (MRI), and bone scintigraphy with ^99m^Tc-diphosphonate. But CT and MRI as morphological imaging techniques are often unable to depict PC foci. For some years now, nuclear medicine methods with functional imaging and detection of metabolic activity, such as hybrid positron emission tomography (PET)/CT or PET/MRI (with various radiopharmaceuticals), have been increasingly used to improve the diagnostic sensitivity and accuracy [5,6,8].

It has been shown that with hybrid imaging the use of a radioligand that binds specifically to the prostate-specific membrane antigen (PSMA) is particularly suitable for the nuclear medical diagnosis and therapy of PC, since malignant prostate cells express PSMA up to 1000 times more strongly than healthy prostate tissue. Thus, PSMA provides an optimal target for diagnosis and therapy of PC lesions. Several PSMA ligands for labeling with ^68^Gallium (^68^Ga) and ^18^Fluor (^18^F) have been developed in recent years and nuclear medicine physicians continue to investigate imaging with [^68^Ga]Ga-PSMA-11 PET/CT (^68^Ga-PSMA) and with [^18^F]PSMA-1007 PET/CT (^18^F-PSMA) [9,10,11,12]. Compared to ^68^Ga-radiolabeled tracers, the ^18^F radionuclides offer advantages in terms of physical properties such as longer half-life and lower positron range with a higher image resolution [9].

The purpose of this study was to evaluate the performance of ^18^F-PSMA and ^68^Ga-PSMA scans in patients with BCR based on calculating the PSA threshold levels for the detection of positive lesions on the PSMA PET/CT and to compare both methods.

## 2. Materials and Methods

### 2.1. Patient Population

This retrospective analysis included 264 patients who had previously been treated by RP or RT. One hundred and thirty-six patients underwent ^68^Ga-PSMA: 128 of these had a BCR by definition and 128 patients underwent ^18^F-PSMA, 106 of whom had a BCR by definition. The data from hybrid imaging, between 2017 and 2021, was retrospectively evaluated. Patients with histopathologically proven primary PC who underwent primary pre-treatment with RP or RT (with or without androgen deprivation therapy) were included in the study. For restaging patients who underwent RP, the EAU guidelines recommend the performance of PSMA PET/CT imaging for PSA values above 0.2 ng/mL [13]. In our study, the recommendation of the EAU was taken into account in the data analysis. Our data was collected from a Practice of Radiology and Nuclear Medicine in Cologne/Germany, named “Praxis im KölnTriangle”. We separately evaluated the data of the patient groups after RP (patient group F-RP for patients who underwent ^18^F-PSMA and patient group Ga-RP who had a ^68^Ga-PSMA) and the groups of patients after RT (patient group F-RT and Ga-RT). RP patients were divided into the following categories: 0.2 to <0.5, 0.5 to <1, 1 to <2, 2 to <5, and ≥5 ng/mL. The categories for RT-patients were: 2 to <5 and ≥5 ng/mL. In this study we used the term “positivity rate” instead of “detection rate” because most of the detected lesions were not histopathologically confirmed. This study was approved by the Ethics Committee of the Medical Association North Rhine (Aekno) (No. 41/2019, approval date: 22 February 2019) and was in accordance with the Helsinki Declaration and the German Medicinal Products Act, AMG § 13.2b. All patients gave their written informed consent for anonymized evaluation and publication of their data.

### 2.2. Imaging Protocol and Analysis

All patients underwent ^18^F-PSMA 90 ± 10 min (whole body) and ^68^Ga-PSMA 60 ± 10 min (whole body) after the intravenous injection of the tracer. Additionally, the included patients underwent late-stage imaging 180 ± 10 min p.i. (pelvic, abdominal, and suspicious regions). PET/CT scans were obtained on a Gemini TF16 PET/CT scanner (Philips Medical Systems, Best, The Netherlands). Attenuation correction of the PET images was performed using a maximum inspiratory venous-phase contrast-enhanced CT scan. PET was performed on a dedicated 3D system (matrix 168 × 168) with an acquisition time of 3 min per bed position (axial field of view 21.8 cm). Emission data were corrected for random, scatter, and decay correction. PET image reconstruction was performed with an ordered-subset expectation maximization algorithm (OSEM) (two iterations and 14 subsets), Gaussian filter with 4.2 mm transaxial resolution at full-width at half maximum. The PET/CT PSMA images were assessed by consensus of two experienced board-certified specialists in nuclear medicine, and two experienced board-certified specialists in radiology, each of them with extensive experience in the interpretation of PET/CT imaging with ^18^F-PSMA and ^68^Ga-PSMA.

### 2.3. Statistical Analysis

The data are presented as means, SD, and/or median and range. To ascertain differences between two groups for continuous metric normally distributed variables, a Student’s *t*-test was performed. A Wilcoxon signed-rank test was carried out to ascertain changes of PSA values. Nominal and ordinal parameters were calculated using the Chi-square test and Pearson correlation. To detect differences in PSA values between the two cohorts (^18^F-PSMA and ^68^Ga-PSMA), we performed a non-parametric Mann–Whitney-U test for independent samples. Moreover, we used PSA categories to perform chi-squared analysis. Additionally, we evaluated a multivariable analysis, in which the detection of a positive scan was a dependent variable, and PSA value, type of PSMA scan (^18^F-PSMA and ^68^Ga-PSMA) and prior therapy with RP versus RT were independent variables. The performance of ^18^F-PSMA and of ^68^Ga-PSMA in relation to the pre-scan PSA values were rated by ROC curve analyses generated by plotting sensitivity versus (vs.) 1-specificity to determine optimal cut-off PSA levels to distinguish positive and negative PET/CT findings. We considered a *p*-value < 0.05 as statistically significant. The statistical analyses were conducted using SPSS version 27.0 (IBM Corporation, Ehningen, Germany).

## 3. Results

### 3.1. Clinical Characteristics of Patients Examined with [^18^F]PSMA-1007 PET/CT

Clinical and pathological characteristics of the 128 included patients examined with ^18^F-PSMA are summarized in Table 1. At the time of the ^18^F-PSMA scan, median PSA was 1.6 ng/mL and range was 0.1–167.1 ng/mL. Sixty-three ^18^F-PSMA patients had a Gleason score (GS, corresponds to the grading method for defining the tumor aggressiveness of PC) [5,13] of 7 (GS 7 includes GS 7a and GS 7b), whereas 9 patients had a GS ≤ 6, 20 ^18^F-PSMA patients had a GS of 8, and 36 patients a GS > 8. Most ^18^F-PSMA patients were primarily treated with RP (F-RP) (84) (Table 1), 78 of whom had a BCR by definition [5].

### 3.2. Clinical Characteristics of Patients Examined with [^68^Ga]Ga-PSMA-11 PET/CT

Of the 136 study patients examined with ^68^Ga-PSMA, the clinical and pathological characteristics are summarized in Table 2. At the time of the ^68^Ga-PSMA scan, the median PSA was 3.2 ng/mL and range was 0.1–170 ng/mL. Eighty-two ^68^Ga-PSMA patients had a GS of 7, whereas 6 patients had a GS ≤ 6. Twenty-seven ^68^Ga-PSMA patients had a GS of 8 and 21 patients had a GS > 8. Most ^68^Ga-PSMA patients were primarily treated with RP (Ga-RP) (92) (Table 2), 89 of whom had a BCR by definition [5].

There was a significant difference in PSA values and PSA categories between the two cohorts (^18^F-PSMA and ^68^Ga-PSMA) (*p* = 0.003, *p* = 0.023) without impact on the positivity rate. A multivariable analysis showed that there was no significant difference (*p* = 0.356) between the two types of PSMA scan (^18^F-PSMA and ^68^Ga-PSMA).

### 3.3. [^18^F]PSMA-1007 PET/CT: Overall Positivity Rate

^18^F-PSMA revealed a positivity rate of 87.5% (112/128 patients) (mean PSA 7.04 ± 18.56 ng/mL). The pre-scan PSA levels differed significantly between the patients with ^18^F-PSMA-positive and ^18^F-PSMA-negative scans (*p* < 0.001). Mean as well as median PSA values of patients with positive scans were significantly higher than those with negative scan results (mean PSA: 7.98 ± 19.70 vs. 0.43 ± 0.30 ng/mL, median PSA: 1.94 vs. 0.39 ng/mL, *p* < 0.001) (Table 3).

### 3.4. [^68^Ga]Ga-PSMA-11 PET/CT: Overall Positivity Rate

The positivity rate of ^68^Ga-PSMA was 88.9% (121 out of 136 patients) (mean PSA 10.4 ± 24.4 ng/mL). Between patients with ^68^Ga-PSMA-positive scans and those with ^68^Ga-PSMA-negative scans, no statistical significance was shown for the differentiation of mean and median PSA (*p* = 0.068) (mean PSA levels of 11.2 ± 25.7 vs. 4.0 ± 5.7 ng/mL, median PSA: 3.7 vs. 1.4 ng/mL, *p* = 0.068) (Table 4).

This retrospective study included 264 patients, 254 that had a BCR by definition [5]. In order to ensure statistical accuracy and a homogeneous patient collective, we considered the two patient groups with different BCR by definition (patient groups BCR patients treated with RP F-RP/Ga-RP and patient groups BCR patients treated with radiotherapy F-RT/Ga-RT) separately.

### 3.5. Positivity Rate with [^18^F]PSMA-1007 PET/CT in Patients Pretreated with Radical Prostatectomy

^18^F-PSMA detected PC lesions in 69 of 78 patients (88%) (mean PSA 8.11 ± 22.75). Significant differences in pre-scan PSA were found between patients with ^18^F-PSMA-positive and ^18^F-PSMA-negative scans (mean PSA: 9.10 ± 24.03 ng/mL vs. 0.51 ± 0.30 ng/mL, median PSA: 1.70 ng/mL vs. 0.40 ng/mL, *p* = 0.002). In patient group F-RP the detection efficacy was 64.7% (11) for PSA levels of 0.2 to <0.5 ng/mL, 94.1% (16), 88.2% (15), 100% (13), and 100% (14) for PSA levels of 0.5 to < 1, 1 to < 2, 2 to < 5, and ≥5 ng/mL, respectively (*p* = 0.009) (Table 5).

The sites of lesions in the ^18^F-PSMA F-RP patient group are shown in Table 5. In 40.6% (28/69) of the patients with a positive scan, local recurrence with or without metastases was evident. Local recurrence without metastases was found in 11 from 69 patients (15.9%). Regional lymph node metastases (LNM) in the pelvis iliac and/or pararectal were shown in 47.8% (33/69).

### 3.6. Positivity Rate with [^68^Ga]Ga-PSMA-11 PET/CT in Patients who Initially Underwent Radical Prostatectomy

In 76 of 89 patients (85%) ^68^Ga-PSMA showed PSMA-avid PC lesions (mean PSA 7.79 ± 21.39). The differentiation between ^68^Ga-PSMA positive and negative scans was not statistically significant (mean PSA: 8.39 ± 23 vs. 4.22 ± 5.99 ng/mL, median PSA: 2.71 vs. 1.40 ng/mL, n.s.). The detection efficacy in the patient group Ga-RP was 75% (12) for PSA levels of 0.2 to <0.5 ng/mL, 86.7% (13), 76.9% (10), 94.4% (17), and 88.9% (24) for PSA levels of 0.5 to <1, 1 to < 2, 2 to < 5, and ≥5 ng/mL, respectively (*p* = 0.108) (Table 6).

In Table 6 the sites of lesions detected in ^68^Ga-PSMA patients (Ga-RP) are shown. Local recurrence with or without metastases was represented in 35.5% (27/76) of patients with positive scans while local recurrence without metastases was shown in 17 from 76 patients (22.4%) and regional LNM were detected in 60.5% (46/76).

### 3.7. Positivity Rate of [^18^F]PSMA-1007 PET/CT in Patients Pretreated with Radiotherapy

All of the 28 BCR patients in the F-RT patient group showed a positive ^18^F-PSMA scan (100%) (mean PSA 9.10 ± 10.16 ng/mL) with 15 patients with PSA levels of 2 to <5 ng/mL and 13 patients with PSA values of ≥5 ng/mL, respectively.

In 82.1% (23/28) of the F-RT patients with a positive ^18^F-PSMA scan, local recurrence with or without metastases was detected, and in 50% (14/28) local recurrence without metastases was revealed.

### 3.8. Positivity Rate of [^68^Ga]Ga-PSMA-11 PET/CT in Patients Pretreated with Radiotherapy

Thirty-eight out of 39 patients had a positive ^68^Ga-PSMA scan (97.4%) (mean PSA 18.76 ± 31.26 ng/mL). For patients treated with RT (Ga-RT), the detection efficacy of ^68^Ga-PSMA was 100% (17) for PSA levels of 2 to <5 ng/mL and 95.5% (21) for PSA levels of ≥5 ng/mL, respectively.

In 71.1% (27/38) of the patients with positive scan results, local recurrence with or without metastases occurred and local recurrence without metastases was detected in 50% (19/38).

### 3.9. PSA Threshold Levels

We assessed the performance of ^18^F-PSMA and ^68^Ga-PSMA in relation to the pre-scan PSA values calculated by the receiver operating characteristic curve (ROC).

#### 3.9.1. PSA Threshold Levels for Patients Treated with Radical Prostatectomy and Examined with [^18^F]PSMA-1007 PET/CT

For predicting positive and negative scans a PSA of 1.08 ng/mL was found to be the best cut-off value by means of ROC analysis (AUC = 0.821; 95% CI 0.710–0.932) in patients pretreated with RP (F-RP).

For patients with a pre-scan PSA below the cut-off (<1.08 ng/mL) the positivity rate of ^18^F-PSMA scan was 76.3% (29/38), and 100% (40/40) with a PSA ≥ 1.08 ng/mL had positive scans (*p* = 0.001) (Table 7).

Distant metastases were noted in 2.6% of the patients with PSA values below cut-off level vs. 32.5% of patients with PSA above cut-off (*p* < 0.001) (Table 7). Multi-metastatic disease was identified in 28.9% below vs. 67.5% above the PSA cut-off levels, respectively (*p* = 0.002) (Table 7).

#### 3.9.2. PSA Threshold Levels for Patients Treated with Radical Prostatectomy and Examined with [^68^Ga]Ga-PSMA-11 PET/CT

For patients that were treated with RP and examined with ^68^Ga-PSMA (Ga-RP) a PSA value of 1.84 ng/mL was best to separate positive and negative scans according to ROC analysis (AUC = 0.588; 95% CI 0.410–0.766).

In patients with a pre-scan PSA value below the cut-off of <1.84 ng/mL, 78.6% (33/42) patients had a positive ^68^Ga-PSMA scan, and in patients with a pre-scan PSA ≥ 1.84 ng/mL positive scan results were noted in 91.5% (43/47) (*p* = 0.085) (Table 8).

Local and distant metastases were determined in 11.9% of the patients with PSA below cut-off vs. 36.2% of patients with PSA levels above cut-off (*p* = 0.014). Multi-metastatic disease appeared in 28.6% vs. 68.1% of the patients with PSA values that were below and above cut-off level (*p* = 0.001), respectively (Table 8).

#### 3.9.3. PSA Threshold Levels for Patients after Initial Radiotherapy Examined with [^18^F]PSMA-1007 PET/CT and [^68^Ga]Ga-PSMA-11 PET/CT

For patients treated with RT (patient group F-RT and Ga-RT), an optimal cut-off could not be obtained by ROC analysis, since only 28 data sets in the patient group F-RT and 39 data sets in the patient group Ga-RT were accessible for the calculation, of which a positive ^18^F-PSMA and ^68^Ga-PSMA scan result was obtained in 28 patients (F-RT) and in 38 patients (Ga-RT).

#### 3.9.4. Subpopulation

The European Association of Urology (EAU) guidelines suggest that PET hybrid imaging with radioactively labeled PSMA ligands could be included as part of the restaging of PC after RP, if the PSA is >0.2 ng/mL and if a therapeutic consequence arises from the PSMA PET/CT findings [13]. In those cases where a PSMA PET/CT scanner is not available, the EAU guidelines suggest to use [^18^F]Fluciclovine PET/CT or [^18^F]Cholin PET/CT when the pre-scan PSA level is >1 ng/mL and if the findings would influence therapeutic decisions [13]. After RP, a PSA of >0.2 ng/mL, confirmed in at least two measurements, indicates a BCR according to German guidelines [5]. The detection rate of PSMA imaging at very low PSA values is of particular clinical interest. Therefore, we looked at the subpopulation data of post-prostatectomized BCR patients with PSA values between 0.2 ng/mL and <1 ng/mL.

In the subpopulation of post-prostatectomy BCR patients with PSA levels between 0.2 ng/mL and <1 ng/mL, in ^18^F-PSMA there was an overall positivity rate of 79.4% (27/34) (*p* = 0.001) with oligo-metastatic disease in 35.3% (12/34) (*p* = 0.002). Local metastases were detected in 58.8% (20/34 (*p* < 0.001).

For the prostatectomized BCR patients with PSA between 0.2 and <1 ng/mL, ^68^Ga-PSMA showed an overall positivity rate of 80.6% (25/31) (*p* = 0.085; n.s.), for oligo-metastatic disease in 22.6% (7/31) (*p* = 0.001) and for local metastases in 38.7% (12/31) (*p* = 0.014).

#### 3.9.5. Maximum Standardized Uptake Value Threshold Levels

To distinguish between patients with low risk (International Society of Urological Pathology/ISUP grade 1; Gleason Score/GS < 7) and favorable intermediate risk PC (ISUP grade 2; GS 7a) vs. unfavorable intermediate risk (ISUP grade 3; GS 7b) and high-risk PC (ISUP grade 4 + 5; GS ≥ 8) we analyzed the data of PSMA positive lesions in the prostate/prostate bed using the ROC curve (generated by plotting sensitivity vs. 1-specificity). In addition, we determined an optimal cut-off with regard to the separation of low and intermediate risk PC from high-risk PC.

Using a maximum standardized uptake value (SUV_max_) of 2.5 as the cut-off level between low and favorable intermediate risk PC vs. unfavorable intermediate and high-risk carcinoma (GS ≤ 7a vs. ≥7b), ^18^F-PSMA the data showed that prostate lesions occurred in 25% of GS ≤ 7a and in 75% of GS ≥ 7b.

By means of ROC analysis a SUV_max_ of 3.35 was found to be a cut-off level to distinguish between GS ≤ 7a vs. ≥7b in ^18^F-PSMA (AUC = 0.605; 95% CI 0.433; 0.778; SD (AUC) = 0.088), but these data were not statistically significant (*p* = 0.209). Additionally, ROC curve analysis showed that a SUV_max_ of 6.85 could distinguish between GS ≤ 7 vs. ≥8 in ^18^F-PSMA (AUC = 0.572; 95% CI 0.429; 0.716; SD (AUC) = 0.073) (*p* = 0.323; n.s.).

^68^Ga-PSMA showed GS ≤ 7a prostate lesions in 31% and GS ≥ 7b prostate lesions in 69% when using a SUV_max_ of 2.5 for the comparison.

ROC curve showed that a SUV_max_ of 3.95 was able to distinguish between GS ≤ 7a and ≥7b in ^68^Ga-PSMA (AUC = 0.512; 95% CI 0.346; 0.677; SD (AUC) = 0.084) (*p* = 0.889; n.s.). In addition, via ROC analysis, a SUV_max_ of 5.65 was found to be an optimal cut-off level to distinguish between GS ≤ 7 vs. ≥8 in ^68^Ga-PSMA (AUC = 0.773; 95% CI 0.623; 0.922; SD (AUC) = 0.076) (*p* = 0.001).

## 4. Discussion

According to EAU guidelines [13] PSMA hybrid imaging is recommended in patients with relapsed PC in several cases. However, it is currently still rated as an “optional” recommendation [13]. According to the authors of the EAU guidelines further study results need to be included before the assessment of PSMA imaging is classified as a “should” or a “mandatory” recommendation. Our study might contribute to elucidate the problem, especially with regard to our comparison of PSMA-targeted radiopharmaceuticals with two different radionuclides.

In our study, the differentiation of PSA between ^18^F-PSMA-positive and ^18^F-PSMA-negative patient scans was statistically significant (*p* < 0.001). Mean PSA values in relation to positive results were significantly higher than those of negative patients scans (mean PSA of 7.98 ± 19.70 ng/mL vs. 0.43 ± 0.30 ng/mL). However, there was no statistical significance using ^68^Ga-PSMA to distinguish mean PSA of 11.2 ± 25.7 vs. 4.0 ± 5.7 ng/mL, *p* = 0.068). The higher median PSA of 3.2 for ^68^Ga-PSMA may be somewhat responsible for the inability to detect a significant association between PSA levels and positive ^68^Ga-PSMA scans, especially since the detection rate of ^68^Ga-PSMA scans at a PSA ≥ 2 ng/mL was quoted as 95% in a recent meta-analysis [14].

**Figure 1:**
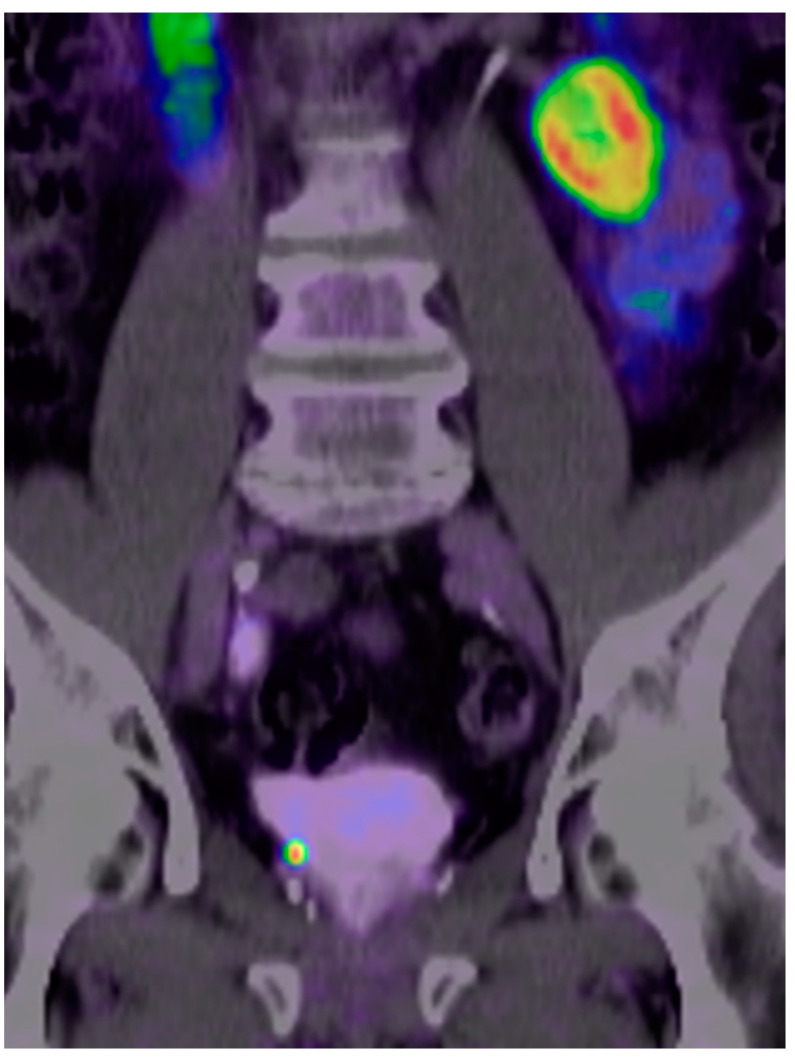
Case study of a prostate cancer (PC) patient who was pretreated with a radical prostatectomy, seminal vesicle removal, and lymphadenectomy (pT3a, pN0 [0/14], cM0, G3) in October 2020 with an initial prostate-specific antigen (PSA) of 6.77 ng/mL. Postoperatively incomplete PSA drop to 0.08 ng/mL. In April 2021 (time of PSMA imaging), the PSA level rose again to 0.197 ng/mL. [^18^F]PSMA-1007 positron-emission tomography/computed tomography (PET/CT) shows a local recurrent PC-lesion near the base on the right in the former seminal vesicle with high prostate-specific membrane antigen (PSMA)-avidity (maximum standardized uptake value/SUV_max_ of 24.3 in the routine images and of 32.4 in the late-stage images) as a correlate of the biochemical recurrence (BCR). (The imaging and data were collected from a Practice of Radiology and Nuclear Medicine in Cologne/Germany, named “Praxis im KölnTriangle”).

The overall high positivity rate of ^18^F-PSMA (87.5%) in our study was slightly lower than the positivity rate of ^68^Ga-PSMA (88.9%) after primary curative PC therapy. However, the difference was higher in the comparison of patients with BCR after RP with a positivity rate of ^18^F-PSMA of 88% while ^68^Ga-PSMA scan patients showed a positivity rate of 85%. Patients with low PC burdens have the best chance of curative results by salvage RT. It is therefore particularly important to obtain meaningful imaging results even at low PSA levels (Figure 1).

For patients after RP the positivity rate with ^18^F-PSMA for PSA values of 0.2 to < 0.5 ng/mL was 64.7% and the corresponding positive rate for ^68^Ga-PSMA was 75%. In the group of patients with PSA levels of 0.5 to <1.0 ng/mL it was 94.1% and 86.7% in ^18^F-PSMA and ^68^Ga-PSMA scans, respectively. A prospective study of Witkowska–Patena et al. [15] in 40 ^18^F-PSMA patients showed substantially lower detection rates with an overall rate of 60% and detection rates of 39% and 55% for PSA values of <0.5 and 0.5 to <1.0 ng/mL, respectively. However, in the prospective Witkowska–Patena study 20% of the 40 patients with BCR after RT were included as well as RT-patients with PSA values up to 2.0 ng/mL [15], while in our study patient data with RP or RT were separately evaluated and we excluded RT-patients with increasing PSA levels < 2.0 ng/mL from our study. Comparable to our patient collective and our results, a study of Giesel et al. with 251 prostatectomized ^18^F-PSMA patients reported a detection rate of 61.5% for PSA of 0.2 to <0.5 ng/mL [16]. While Rahbar et al. evaluated a ^18^F-PSMA detection rate of 85.7% for patients with PSA levels up to <0.5 ng/mL and 88.9% in the patient group with PSA between 0.5 and 1.0 ng/mL [9]. Dietlein et al. compared the PSA-stratified performance of ^18^F- and ^68^Ga-PSMA scans in BCR patients which showed an advantage for ^18^F-PSMA at PSA values of 0.5 to 3.5 μg/L with a sensitivity of 88% for ^18^F-PSMA vs. 66% for ^68^Ga-PSMA [17]. When considering the entire patient population included in the Dietlein study, the results of both scanning methods were found to be similar [17].

Recent studies show that ^18^F-PSMA as well as ^68^Ga-PSMA tumor burden significantly correlates with PSA values [16,18,19]. For prostatectomized ^18^F-PSMA patients in our study, metastases were detected in 84.1%, whereas in the ^68^Ga-PSMA group metastatic disease occurred in 76.3%. PSA-stratified loco-regional LNM were present in 29.4% (patient group F-RP) and in 31.3% (Ga-RP) for PSA values of 0.2 to <0.5 ng/mL. Our results in F-RP are in line with a study of 251 BCR patients after RP that reported local LNM in 26.2% for PSA levels between 0.2 and <0.5 ng/mL [16]. Furthermore, bone metastases were detected in 24.6% in the same PSA-group [16]. However, our PSMA-positive results in the patient group Ga-RP do not show a significant steady increase between the patients with PSA values < 0.5 ng/mL and those with PSA ≥ 5.0 ng/mL in contrast to other studies [19]. This is probably due to the low number of cases belonging to the respective groups (e.g., 16 patients with PSA values < 0.5 ng/mL and 27 patients with PSA ≥ 5.0 ng/mL). But Afshar–Oromieh et al. [10] also demonstrated in a large ^68^Ga-PSMA patient cohort that a steady increase of PSA did not always correlate with an increase in PC detection, similar to our results. The authors suggested that PSMA-negative scans may be due to a dedifferentiation of the tumor or other reasons such as a too small tumor size below the resolution rate of the PET scanner or that the PC-lesions are too close to the urinary bladder to be detected [10]. Dietlein et al. found an interesting discrepancy in the ^18^F-PSMA detection of loco-regional lesions and skeletal metastases with a high number of non-specific focal bone marrow uptake without morphologic correlates on CT. A supplementary skeletal MRI was also unable to confirm any malignancy [20]. The authors concluded that ^18^F-PSMA is very helpful in the interpretation of loco-regional PC lesions, but for bone metastases, additional verification such as PET/MRI is required and other PSMA PET tracers such as ^68^Ga-PSMA should be preferred [20]. Another study that focused primarily on pitfalls and varying interpretation of results comparing ^18^F-PSMA with ^68^Ga-PSMA, found a substantially higher rate of visually verifiable PSMA-avid lesions with an intensified SUV_max_ which was ascribed to a benign emergence in case of ^18^F-PSMA than of ^68^Ga-PSMA [21]. On the other hand, the PSMA PET/CT results in this study were comparable with regard to the detection rate [21]. Recent case reports and reviews have described the pitfalls of PSMA-avid benign lesions in PSMA PET; for example, in the ganglia [21]. However, Rauscher et al. found a discrepancy between the number of PSMA-positive benign lesions in ^18^F-PSMA compared to ^68^Ga-PSMA and suggested that it could be due to the different properties such as lower positron energy of ^18^F, resulting in a shorter positron range and a higher positron yield with an improved spatial resolution [21,22]. In a preclinical intra-individual dynamic comparison of ^18^F-PSMA and ^68^Ga-PSMA in mice the tracer uptake parameters were considerably higher in ^18^F-PSMA [22]. We did not verify these findings in our clinical study, although it could have been explained by the different acquisition protocols with different tracer uptake time periods (recording times after tracer injection: ^18^F-PSMA 90 ± 10 min post-injectionem (p.i.) vs. ^68^Ga-PSMA 60 ± 10 min p.i.). Consistent with the conclusion of the Rauscher research group, the PSMA images (including the ^18^F-PSMA cases) in our study were interpreted exclusively by highly experienced specialists in both functional imaging (PET) and morphological imaging (MRI, especially multiparametric MRI). Additionally, all suspicious lesions were verified by late-stage PSMA-PET/CT imaging and malignancy was confirmed or excluded. In still unclear cases, additional MRI recordings were made. For this reason, all false positive foci could be recognized as benign and accordingly correctly assessed in the findings.

Recent studies demonstrated significant positive correlations between ^18^F-PSMA as well as ^68^Ga-PSMA uptake with serum PSA values and tumor aggressiveness (expressed as GS) [19,23,24]. Using an SUV_max_ of 2.5 with regard to separate low and favorable intermediate risk PC vs. unfavorable intermediate and high-risk PC (GS ≤ 7a vs. ≥7b), ^18^F-PSMA showed that 25% GS ≤ 7a prostatic lesions and 75% GS ≥ 7b PC lesions occurred in the prostate bed in our study, while ^68^Ga-PSMA showed GS ≤ 7a and GS ≥ 7b prostatic bed lesions in 31% and 69% when using a SUV_max_ cut-off of 2.5. Furthermore, using ROC analysis we determined optimal cut-off levels for the separation of low and intermediate risk PC from high-risk PC. However, only one case showed statistical significance with a SUV_max_ cut-off of 5.65 to distinguish between GS ≤ 7 vs. ≥8 in ^68^Ga-PSMA (AUC = 0.773; 95% CI 0.623; 0.922; SD (AUC) = 0.076) (*p* = 0.001). Our results are in line with other studies [19,24].

To determine the optimal time for the performance of ^18^F-PSMA and ^68^Ga-PSMA for restaging, we identified optimal PSA threshold levels for detecting PSMA-positive findings. Limitations of the present study are that we could not calculate any PSA kinetic thresholds for PSA doubling time or PSA velocity [19] because the PSA course values were not available for all data sets and that most of the detected lesions were not histopathologically confirmed (In case of BCR, when biopsy or surgery of PSMA-avid lesions were not possible or considered too invasive for the patients, e.g., bone metastases, we rated the increase of PSA before therapy and decrease after an ensuing therapy or results of follow-up imaging beyond the histopathological results as a tumor confirmation and marker). For ^18^F-PSMA a PSA value of 1.08 ng/mL was found to be the optimal cut-off for predicting positive and negative scans in prostatectomized patients. In patients with a pre-scan PSA < 1.08 ng/mL, the positivity rate was 76.3%, whereas patients with a PSA ≥ 1.08 ng/mL showed a scan positivity in 100% (*p* = 0.001). For multi-metastatic disease the rates were 28.9% vs. 67.5% (*p* = 0.002). On the other hand, for ^68^Ga-PSMA patients primarily treated with RP, the calculated optimal cut-off was 1.84 ng/mL with positivity rates of 78.6% and 91.5% (<1.84 and ≥ 1.84 ng/mL, *p* = 0.085). The higher PSA threshold value of ^68^Ga-PSMA compared to ^18^F-PSMA could possibly be related to the patient population. Both collectives are consecutive patients. However, the increasing number of referrals for prostate patients by urologists has only occurred in recent years. At the beginning of PSMA imaging, the number of assignments were increased when the PSA values were already higher. Only with higher acceptance of PSMA PET/CT-results among urologists, patients with low PSA levels were also referred in the early stages of the recurrent disease. At the same time, PSMA imaging began at ^18^F. In our study, the rates for multi-metastatic disease in the ^68^Ga-PSMA group for patients with PSA values below and above the calculated threshold were 28.6% vs. 68.1% (*p* = 0.001). Overall, the findings obtained with ^18^F-PSMA and ^68^Ga-PSMA were similar as also reported by other authors [25,26]. An interesting aspect from our point of view is that a significant subgroup of oligo-metastatic disease could be identified below the threshold of both methods which may be suitable as a selection criterion for targeted therapy. However, the importance of targeted therapy in the context of urological studies is discussed, in particular whether the targeted therapy of oligo-metastatic prostate disease has an effect on overall survival. However, we would like to put up for discussion that decisions on therapy management for patients with PSA relapse should not only be based on the evidence of positive findings on the restaging of PSMA-PET/CT, but in particular on the effects on overall survival. Our study has limitations. Here, we used real world data to indicate a threshold for pre-scan PSA whereas many centers implement restaging for all patients with elevated PSA values. Although a BCR after RP is only taken into account from a PSA value of 0.2 ng/mL according to the German guidelines [5] and PSMA PET/CT imaging is recommended for patients with a PSA value > 0.2 ng/mL according to the EAU guidelines [13], the patient group with pre-scan PSA values below 0.2 ng/mL could also be included in determining the effects on the outcome [27]. The alternative approach to the link between PSA and PSMA hybrid imaging could be to assess the performance of the PSMA PET/CT in terms of a pre-scan PSA level that clinicians consider best for the outcome of restaging patients. A recent study reported the overall survival in relation to clinically goal of a pretreatment PSA of <0.5 ng/mL before salvage therapy as a threshold to separate patients with BCR into two groups with a marked 20% difference in 5-year overall survival after the PSMA PET/CT [28]. Thus, the optimal pre-scan PSA value for overall survival could potentially be clearly different from the ROC-based optimal PSA level considering the lower PSA limit [13]. It should also be considered that there may be micrometastases in spite of PSMA-negative scans. Studies in this regard are currently being carried out.

## 5. Conclusions

Our study underscores the important diagnostic role of PSMA hybrid imaging in the detection of PC recurrence. Both ^18^F-PSMA and ^68^Ga-PSMA demonstrated high positivity rates in PC restaging with comparable results. Only the identified PSA threshold values for the presence of PSMA-avid PC lesions in BCR patients after RP showed a clearer distinction between positive and negative scan findings with ^18^F-PSMA compared to ^68^Ga-PSMA. Below the threshold value of both methods, a significant subgroup of oligo-metastatic disease could be identified that may be suitable as a selection criterion for targeted therapy (as opposed to systemic therapy). Since there was no clear diagnostic advantage for ^18^F-PSMA vs. ^68^Ga-PSMA, administrative aspects and selection criteria such as availability, approval by health authorities, legal requirements (not addressed in the present study), as well as theranostic strategy and examination time may contribute to the choice of PSMA hybrid imaging in restaging of PC.

## Figures and Tables

**Table 1:** Characteristics of 128 patients examined with restaging [^18^F]PSMA-1007 PET/CT.

Characteristics (*n*)	Parameters
Number of [^18^F]PSMA-1007 PET/CT patients	128
Age (y)	
- Median	69
- Range	51–96
Mean ± SD	69.3 ± 8.8
Gleason Score	
- ≤6 (low risk + grade group 1)	9
- 7 (intermediate risk + grade group 2 + 3)	63
- 8 (high risk + grade group 4)	20
- >8 (high risk + grade group 5)	36
PSA (ng/mL)	
- Median	1.6
- Range	0.1–167.1
- Mean ± SD	7.0 ± 18.6
Pretreatment of primary tumor	
- Surgery (radical prostatectomy)	84
- Radiotherapy	44
Further treatment	
- Androgen deprivation therapy	74
Positivity rate	
- PET/CT positive patients/total	112/128

Abbreviations: PSA, prostate-specific antigen; SD, standard deviation; *n*, number of patients; y, year.

**Table 2:** Characteristics of 136 patients examined with restaging [^68^Ga]Ga-PSMA-11 PET/CT.

Characteristics (*n*)	Parameters
Number of [68Ga]Ga-PSMA-11 PET/CT patients	136
Age (y)	
- Median	70
- Range	49–94
- Mean ± SD	69.2 ± 8.3
Gleason Score	
- ≤6 (low risk + grade group 1)	6
- 7 (intermediate risk + grade group 2 + 3)	82
- 8 (high risk + grade group 4)	27
- >8 (high risk + grade group 5)	21
PSA (ng/mL)	
- Median	3.2
- Range	0.1–170
- Mean ± SD	10.4 ± 24.4
Pretreatment of primary tumor	
- Surgery (radical prostatectomy)	92
- Radiotherapy	44
Further treatment	
- Androgen deprivation therapy	60
Positivity rate	
- PET/CT positive patients/total	121/136

Abbreviations: PSA, prostate-specific antigen; SD, standard deviation; *n*, number of patients; y, year.

**Table 3:** Positive vs. negative [^18^F]PSMA-1007 PET/CT results in relation to PSA.

PSA (ng/mL)	PET/CT Results		
	Positive (112/128)	*p*-Value	Negative (16/128)
Mean ± SD	7.98 ± 19.70		0.43 ± 0.30
Median (range)	1.94 (0.10–167)		0.39 (0.13–1.00)
		*p* < 0.001	

Abbreviations: PSA, prostate-specific antigen; SD, standard deviation; *p*-value < 0.05 is considered significant.

**Table 4:** Positive vs. negative [^68^Ga]Ga-PSMA-11 PET/CT results in relation to PSA.

PSA (ng/mL)	PET/CT Results		
	Positive (121/136)	*p*-Value	Negative (15/136)
Mean ± SD	11.2 ± 25.7		4.0 ± 5.7
Median (range)	3.7 (0.08–170)		1.4 (0.14–18)
		*p* = 0.068	

Abbreviations: PSA, prostate-specific antigen; SD, standard deviation; *p*-value < 0.05 is considered significant.

**Table 5:** Position of detectable cancerous lesions in [^18^F]PSMA-1007 PET/CT patients (pretreated with radical prostatectomy/F-RP) with relation to PSA.

PSA (ng/mL)	0.2 -< 0.5	0.5 -< 1.0	1.0 -< 2.0	2.0 -< 5.0	≥5.0	Chi^2^, *r*
Number (x/78) patient group F-RP	17	17	17	13	14	
PET/CT positive	11/64.7%	16/94.1%	15/88.2%	13/100%	14/100%	*p* = 0.009 *r* = 0.340
Local recurrence without metastases	5	1	3	2	0	
Local recurrence with local metastases (regional metastases in the pelvis: iliac and/or pararectal)	0	1	1	1	1	
Local metastases without local recurrence	4	3	2	0	0	
Local recurrence with distant metastases (within 2 regions)	0	1	0	0	1	
Distant metastases (within 2 regions) without local recurrence	1	2	1	1	0	
Local recurrence with multiple metastases	0	2	3	4	2	
Multiple metastases without local recurrence	1	6	5	5	10	
Number of metastases:						*p* = 0.001 *r* = 0.522
Single metastases	5	7	4	2	2	
Multiple metastases	1	8	8	9	12	
Lymph node metastases (LNM)	5	12	10	9	9	*p* = 0.103 *r* = 0.200
Site of LNM:						*p* = 0.121 * r = 0.333
Local LNM (regional LNM in the pelvis: iliac and/or pararectal)	5	10	8	6	4	
Distant LNM (extrapelvic LNM: retroperitoneal and/or above the iliac bifurcation)	0	1	1	0	1	
Local + distant LNM	0	1	1	3	4	
Bone metastases	1	3	4	4	9	*p* = 0.007 * r = 0.407
Visceral metastases	0	0	0	0	0	

* Fisher exact test. Abbreviations: PSA, prostate-specific antigen; LNM, lymph node metastases; *p*-value < 0.05 is considered significant; *r*, Pearson correlation coefficient.

**Table 6:** Position of detectable cancerous lesions in [^68^Ga]Ga-PSMA-11 PET/CT patients (pretreated with radical prostatectomy/Ga-RP) with relation to PSA.

PSA (ng/mL)	0.2 -< 0.5	0.5 -< 1.0	1.0 -< 2.0	2.0 -< 5.0	≥5.0	Chi^2^, *r*
Number (x/89) patient group Ga-RP	16	15	13	18	27	
PET/CT positive	12/75%	13/86.7%	10/76.9%	17/94.4%	24/88.9%	*p* = 0.108 *r* = 0.142
Local recurrence without metastases	4	5	3	5	0	
Local recurrence with local metastases (regional metastases in the pelvis: iliac and/or pararectal)	0	0	0	0	0	
Local metastases without local recurrence	2	1	0	1	1	
Local recurrence with distant metastases (within 2 regions)	0	0	1	0	0	
Distant metastases (within 2 regions) without local recurrence	2	2	2	1	2	
Local recurrence with multiple metastases	0	1	2	2	4	
Multiple metastases without local recurrence	4	4	2	8	17	
Number of metastases:						*p* = 0.018 * r = 0.386
Single metastases	4	3	3	2	3	
Multiple metastases	4	5	4	10	21	
Lymph node metastases (LNM)	7	6	5	8	20	*p* = 0.094 *r* = 0.227
Site of LNM:						*p* = 0.376 * r = 0.226
Local LNM (regional LNM in the pelvis: iliac and/or pararectal)	5	4	3	5	12	
Distant LNM (extrapelvic LNM: retroperitoneal and/or above the iliac bifurcation)	1	0	2	1	2	
Local + distant LNM	1	2	0	2	6	
Bone metastases	2	2	3	4	12	*p* = 0.101 * r = 0.267
Visceral metastases	0	0	1	0	0	

* Fisher exact test. Abbreviations: PSA, prostate-specific antigen; LNM, lymph node metastases; *p*-value < 0.05 is considered significant; *r*, Pearson correlation coefficient.

**Table 7:** [^18^F]PSMA-1007 PET/CT in 78 patients initially treated with radical prostatectomy (F-RP): subgroups of patients according to the number of PSMA-positive lesions categorized by pre-scan PSA threshold 1.08 ng/mL.

**PSA Range (ng/mL)**	**Overall** **Positivity**	***p*/*r*-Value**	**Oligo-Metastatic**	**Multi-Metastatic**	***p*/*r*-Value**	
<1.08 (38)	29 (76.3%)		12 (31.6%)	11 (28.9%)		
≥1.08 (40)	40 (100%)		8 (20.0%)	27 (67.5%)		
Total (78)	69 (88.4%)	*p* = 0.001 *r* = 0.371	20 (25.6%)	38 (48.7%)	*p* = 0.002 *r* = 0.394	
**PSA Range (ng/mL)**	**Local** **Recurrence**	***p*/*r*-Value**	**Local Metastases**	**Distant** **Metastases**	**Local + Distant Metastases**	***p*/*r*-Value**
<1.08 (38)	11 (28.9%)		22 (57.9%)	1 (2.6%)	0 (0%)	
≥1.08 (40)	17 (42.5%)		20 (50.0%)	13 (32.5%)	2 (5.0%)	
Total (78)	28 (35.9%)	*p* = 0.212 *r* = 0.141	42 (53.8%)	14 (17.9%)	2 (2.6%)	*p* < 0.001 *r* = 0.456

Abbreviations: PSA, prostate-specific antigen; *p*-value < 0.05 is considered significant.

**Table 8:** [^68^Ga]Ga-PSMA-11 PET/CT in 89 patients initially treated with radical prostatectomy (Ga-RP): subgroups of patients according to the number of PSMA-positive lesions categorized by pre-scan threshold 1.84 ng/mL.

**PSA Range (ng/mL)**	**Overall Positivity**	***p*/*r*-Value**	**Oligo-Metastatic**	**Multi-Metastatic**	***p*/*r*-Value**	
<1.84 (42)	33 (78.6%)		10 (23.8%)	12 (28.6%)		
≥1.84 (47)	43 (91.5%)		5 (10.6%)	32 (68.1%)		
Total (89)	76 (85.4%)	*p* = 0.085 *r* = 0.183	15 (16.9%)	44 (49.4%)	*p* = 0.001 *r* = 0.366	
**PSA Range (ng/mL)**	**Local Recurrence**	***p*/*r*-Value**	**Local Metastases**	**Distant Metastases**	**Local + Distant Metastases**	***p/r*-Value**
<1.84 (42)	14 (33.3%)		10 (23.8%)	7 (16.7%)	5 (11.9%)	
≥1.84 (47)	13 (27.7%)		9 (19.1%)	11 (23.4%)	17 (36.2%)	
Total (89)	27 (30.3%)	*p* = 0.561 *r* = −0.062	19 (21.3%)	18 (20.2%)	22 (46.8%)	*p* = 0.014 *r* = 0.344

Abbreviations: PSA, prostate-specific antigen; *p*-value < 0.05 is considered significant.

## Data Availability

The datasets used and/or analyzed during the current study are available from the Practice of Radiology and Nuclear Medicine in Cologne/Germany, named “Praxis im KölnTriangle” upon reasonable request.

## References

[1] Ferlay J., Colombet M., Soerjomataram I., Dyba T., Randi G., Bettio M., Gavin A., Visser O., Bray F. (2018). Cancer incidence and mortality patterns in Europe: Estimates for 40 countries and 25 major cancers in 2018. Eur. J. Cancer.

[2] Pilleron S., Sarfati D., Janssen-Heijnen M., Vignat J., Ferlay J., Bray F., Soerjomataram I. (2019). Global Cancer Incidence in Older Adults, 2012 and 2035: A population-based study. Int. J. Cancer.

[3] Rider J.R., Sandin F., Andrén O., Wiklund P., Hugosson J., Stattin P. (2013). Long-term outcomes among noncuratively treated men according to prostate cancer risk category in a nationwide, population-based study. Eur. Urol..

[4] Ruigrok E.A.M., van Weerden W.M., Nonnekens J., de Jong M. (2019). The Future of PSMA-Targeted Radionuclide Therapy: An Overview of Recent Preclinical Research. Pharmaceutics.

[5] Leitlinienprogramm Onkologie (Deutsche Krebsgesellschaft, Deutsche Krebshilfe, AWMF): S3-Leitlinie Prostatakarzinom, Langversion 6.2, 2021, AWMF Registernummer: 043/022OL. http://www.leitlinienprogramm-onkologie.de/leitlinien/prostatakarzinom/.

[6] Fanti S., Minozzi S., Antoch G., Banks I., Briganti A., Carrio I., Chiti A., Clarke N., Eiber M., De Bono J. (2018). Consensus on molecular imaging and theranostics in prostate cancer. Lancet Oncol..

[7] Tangel M.R., Rastinehad A.R. (2018). Advances in prostate cancer imaging. F1000 Res..

[8] Pijoan J.M. (2006). Diagnostic methodology for the biochemical recurrence of prostate cancer after radiotherapy. Arch. Esp. Urol..

[9] Treglia G., Annunziata S., Pizzuto D.A., Giovanella L., Prior J.O., Ceriani L. (2019). Detection Rate of 18F-Labeled PSMA PET/CT in Biochemical Recurrent Prostate Cancer: A Systematic Review and a Meta-Analysis. Cancers.

[10] Afshar-Oromieh A., Holland-Letz T., Giesel F.L., Kratochwil C., Mier W., Haufe S., Debus N., Eder M., Eisenhut M., Schäfer M. (2017). Diagnostic performance of 68Ga-PSMA-11 (HBED-CC) PET/CT in patients with recurrent prostate cancer: Evaluation in 1007 patients. Eur. J. Nucl. Med. Mol. Imaging.

[11] Coenen H.H., Gee A.D., Adam M., Antoni G., Cutler C.S., Fujibayashi Y., Jeong J.M., Mach R.H., Mindt T.L., Pike V.W. (2019). Open letter to journal editors on: International Consensus Radiochemistry Nomenclature Guidelines. EJNMMI Radiopharm. Chem..

[12] Von Eyben F.E., Kairemo K., Paller C., Hoffmann M.A., Paganelli G., Virgolini I., Roviello G. (2021). 177Lu-PSMA Radioligand Therapy Is Favorable as Third-Line Treatment of Patients with Metastatic Castration-Resistant Prostate Cancer. A Systematic Review and Network Meta-Analysis of Randomized Controlled Trials. Biomedicines.

[13] (2021). EAU Guidelines. Proceedings of the EAU Annual Congress.

[14] Perera M., Papa N., Roberts M., Williams M., Udovicich C., Vela I., Christidis D., Bolton D., Hofman M.S., Lawrentschuk N. (2020). Gallium-68 Prostate-specific Membrane Antigen Positron Emission Tomography in Advanced Prostate Cancer-Updated Diagnostic Utility, Sensitivity, Specificity, and Distribution of Prostate-specific Membrane Antigen-avid Lesions: A Systematic Review and Meta-analysis. Eur. Urol..

[15] Witkowska-Patena E., Giżewska A., Dziuk M., Miśko J., Budzyńska A., Walęcka-Mazur A. (2020). Diagnostic performance of 18F-PSMA-1007 PET/CT in biochemically relapsed patients with prostate cancer with PSA levels ≤ 2.0 ng/mL. Prostate Cancer Prostatic Dis..

[16] Giesel F.L., Knorr K., Spohn F., Will L., Maurer T., Flechsig P., Neels O., Schiller K., Amaral H., Weber W.A. (2019). Detection Efficacy of 18F-PSMA-1007 PET/CT in 251 Patients with Biochemical Recurrence of Prostate Cancer After Radical Prostatectomy. J. Nucl. Med..

[17] Dietlein F., Kobe C., Neubauer S., Schmidt M., Stockter S., Fischer T., Schomäcker K., Heidenreich A., Zlatopolskiy B.D., Neumaier B. (2017). PSA-Stratified Performance of 18F- and 68Ga-PSMA PET in Patients with Biochemical Recurrence of Prostate Cancer. J. Nucl. Med..

[18] Schmidkonz C., Cordes M., Schmidt D., Bäuerle T., Goetz T.I., Beck M., Prante O., Cavallaro A., Uder M., Wullich B. (2018). 68Ga-PSMA-11 PET/CT-derived metabolic parameters for determination of whole-body tumor burden and treatment response in prostate cancer. Eur. J. Nucl. Med. Mol. Imaging.

[19] Hoffmann M.A., Buchholz H.G., Wieler H.J., Miederer M., Rosar F., Fischer N., Müller-Hübenthal J., Trampert L., Pektor S., Schreckenberger M. (2020). PSA and PSA Kinetics Thresholds for the Presence of 68Ga-PSMA-11 PET/CT-Detectable Lesions in Patients With Biochemical Recurrent Prostate Cancer. Cancers.

[20] Dietlein F., Kobe C., Hohberg M., Zlatopolskiy B.D., Krapf P., Endepols H., Täger P., Hammes J., Heidenreich A., Persigehl T. (2020). Intraindividual Comparison of 18F-PSMA-1007 with Renally Excreted PSMA Ligands for PSMA PET Imaging in Patients with Relapsed Prostate Cancer. J. Nucl. Med..

[21] Rauscher I., Krönke M., König M., Gafita A., Maurer T., Horn T., Schiller K., Weber W., Eiber M. (2020). Matched-Pair Comparison of 68Ga-PSMA-11 PET/CT and 18F-PSMA-1007 PET/CT: Frequency of Pitfalls and Detection Efficacy in Biochemical Recurrence After Radical Prostatectomy. J. Nucl. Med..

[22] Piron S., Verhoeven J., Descamps B., Kersemans K., De Man K., Van Laeken N., Pieters L., Vral A., Vanhove C., De Vos F. (2020). Intra-individual dynamic comparison of 18F-PSMA-11 and 68Ga-PSMA-11 in LNCaP xenograft bearing mice. Sci. Rep..

[23] Hoffmann M.A., Buchholz H.G., Wieler H.J., Rosar F., Miederer M., Fischer N., Schreckenberger M. (2020). Dual-Time Point [68Ga]Ga-PSMA-11 PET/CT Hybrid Imaging for Staging and Restaging of Prostate Cancer. Cancers.

[24] Hong J.J., Liu B.L., Wang Z.Q., Tang K., Ji X.W., Yin W.W., Lin J., Zheng X.W. (2020). The value of 18F-PSMA-1007 PET/CT in identifying non-metastatic high-risk prostate cancer. EJNMMI Res..

[25] Hoberück S., Löck S., Borkowetz A., Sommer U., Winzer R., Zöphel K., Fedders D., Michler E., Kotzerke J., Kopka K. (2021). Intraindividual comparison of [68 Ga]-Ga-PSMA-11 and [18F]-F-PSMA-1007 in prostate cancer patients: A retrospective single-center analysis. EJNMMI Res..

[26] Emmett L., Ende J., Amin A., Sheehan-Dare G., Cusick T. (2021). Pilot Trial Comparing the Performance of 68Ga-PSMA-11 PET/CT to 18F-PSMA-1007 PET/CT in the Detection of Prostate Cancer Recurrence in Men with Rising PSA Following Radical Prostatectomy. J. Radiol. Med. Imaging.

[27] von Eyben F.E., Bauman G., Mateo J., Fizazi K., Gillessen S., Heidenreich A., Perez-Lopez R., Oyen W.J.G., Shore N., Smith M. (2019). Managing Nonmetastatic Castration-resistant Prostate Cancer. Eur. Urol..

[28] von Eyben R., Hoffmann M.A., Kapp D.S., Soydal C., Uprimny C., Virgolini I., Tuncel M., Gauthé M., von Eyben F.E. (2022). Quality Goal for Salvage Treatment for Patients with Prostate Cancer at Prostate-specific Antigen Relapse. Eur. Urol. Oncol..

